# Enhancing monitoring and evaluation of digital health interventions in sub-Saharan Africa: big data, mHealth, and dashboards

**DOI:** 10.7189/jogh.15.03013

**Published:** 2025-03-21

**Authors:** Oche Joseph Otorkpa, Joseph Omeiza Alao, Abiodun Paul Olaiya

**Affiliations:** 1 Department of Public Health, Faculty of Health Sciences, National Open University of Nigeria, Lokoja, Nigeria; 2 Department of Physics, Air Force Institute of Technology, Kaduna, Nigeria; 3 Department of Public Health, School of Public Health, Texila American University, Georgetown, Guyana

## Abstract

Digital health interventions based on digital and mobile technologies are essential for advancing healthcare in sub-Saharan Africa. However, challenges with their effective monitoring and evaluation (M&E) continue to impact their scalability and sustainability. Here we propose a set of innovative strategies to strengthen M&E frameworks, including integrating big data analytics and artificial intelligence for real-time impact assessment, leveraging mHealth platforms for enhanced data collection and stakeholder engagement, and developing interactive digital dashboards for transparent decision-making. These strategies aim to optimise digital health interventions implementation and evaluation, addressing critical healthcare access and outcome challenges exacerbated by a shortage of healthcare professionals in the region. By enhancing M&E capabilities, these approaches maximise the impact of digital health technologies in improving public health across sub-Saharan Africa.

Digital health interventions (DHIs) refer to the application of digital and mobile technologies to enhance health system service delivery. In recent years, advancements in computing, genomics, and artificial intelligence have increasingly been incorporated into the domain of digital health, defined by the World Health Organization (WHO) global strategy for 2020–25 as ‘the field of knowledge and practice associated with the development and use of digital technologies to improve health’ [1].

Monitoring and evaluation (M&E) of health interventions are critical for assessing the efficiency, effectiveness, and impact of health programmes. Monitoring involves the systematic and continuous collection, analysis, and use of data to track the progress of an intervention against pre-defined objectives and indicators. It focusses on the day-to-day activities and outputs, ensuring that the programme is being implemented as planned and identifying any issues that may arise during implementation [2]. Evaluation, on the other hand, is the systematic assessment of the relevance, effectiveness, efficiency, impact, and sustainability of an intervention. It is typically conducted at specific points in time, such as mid-term or upon completion of the intervention, and provides an in-depth analysis of whether the intervention achieved its goals and why. Evaluations help determine the overall value and benefits of the programme, guide decision-making for future interventions, and ensure accountability to stakeholders [3].

Key components of M&E include indicators, data collection methods, baseline data, monitoring systems, evaluation design, and reporting and dissemination. Indicators are measurable markers of progress, either quantitative or qualitative. Data collection methods, such as surveys and health records, ensure accurate M&E. Baseline data provides a reference point for measuring changes. Monitoring systems track progress using tools like management information systems. Evaluation design, which includes various methodological frameworks, helps attribute changes to an intervention. Reporting and dissemination involve compiling and communicating findings to stakeholders, ensuring transparency and informed decision-making. All these components are vital when assessing the impact of DHIs [4].

Studies indicate that Africa, particularly sub-Saharan Africa, boasts a diverse array of digital health solutions. However, the region faces significant challenges in the coordination, integration, scalability, sustainability, and equitable distribution of investments in digital health [5]

For example, available data indicate that Africa struggles with poor M&E of health systems and interventions, with far-reaching implications for the health and well-being of its populations [6]. Despite accounting for 11–13% of the world's population, the continent bears 24% of the global disease burden; despite this, investment in healthcare in sub-Saharan Africa accounts for less than 1% of global health expenditure, making the deployment of effective M&E a major necessity in order to ensure the proper utilisation of scarce resources [7].

The rapid adoption of DHIs across sub-Saharan Africa represents a transformative shift in the delivery of healthcare [8]. In recent years, many countries in the region have implemented comprehensive digital health strategies, which encompass the deployment of telemedicine, mHealth (*i.e. *care relying on mobile devices), and eHealth technologies to enhance service coverage and improve care quality, particularly for underserved populations. There is promising evidence of progress in various initiatives designed to augment the utilisation of digital health tools for public health if (M&E) frameworks are adopted and implemented appropriately [9].

## STRATEGIES

Considering the current financial constraints, poor state of health systems efficiency in the region and rapidly dwindling number of health worker, there is an urgent need to adopt robust and innovative M&E frameworks to ensure these digital innovations achieve their full potential interns of effectiveness, scalability and sustainability [10]. Here, we propose three unique strategies to enhance the effectiveness of these processes.

The integration of big data analytics and responsible application of artificial intelligence (AI) can significantly improve the M&E of DHIs. By analysing large volumes of health data, AI can identify patterns and predict outcomes with greater accuracy, provided it is used ethically and responsibly. This allows for real-time monitoring of intervention impacts, enabling stakeholders to make data-driven decisions when adjusting programmes. The predictive power of AI can also forecast future health trends, thereby informing proactive intervention planning. The automation of data analysis further reduces the burden on human resources, allowing healthcare professionals to focus on direct patient care and other critical tasks.

Second, (mHealth) platforms can be utilised to collect real-time data from patients and healthcare providers. This approach not only increases the accuracy and timeliness of data, but also enhances the engagement of stakeholders. Mobile surveys, SMS-based reporting, and app-based data collection tools offer valuable insights into the effectiveness and challenges of DHIs. This continuous flow of data facilitates timely interventions, such as adjusting treatment plans or addressing emerging health issues. Additionally, mHealth platforms can support remote patient monitoring, particularly in regions with limited access to healthcare facilities, thereby improving healthcare delivery and outcomes in underserved populations.

The creation of interactive digital dashboards that consolidate various data streams into a single platform can enhance the visualisation and interpretation of M&E data. These dashboards can be utilised by healthcare providers, policymakers, and researchers to track progress, identify trends, and make informed decisions. Features such as geospatial mapping and real-time data updates provide a comprehensive overview of intervention impacts, with the latter ensuring that decision-makers have access to the most up-to-date information, which is crucial for responding to rapidly changing health scenarios. Interactive dashboards also facilitate the dissemination of information to a wider audience, promoting transparency and accountability in health programmes.

The integration of these strategies – big data analytics, mHealth platforms, and interactive dashboards – can transform the M&E landscape for DHIs. Big data analytics and AI enable the processing of vast amounts of data to uncover hidden patterns and predict future health trends. This enhances the ability to monitor and evaluate health programmes in real-time, leading to more effective and timely interventions. mHealth platforms provide a direct link between patients and healthcare providers, facilitating continuous data collection and engagement. This type of real-time data collection is essential for capturing the dynamic nature of health interventions and ensuring that responses are swift and appropriate. Interactive dashboards, meanwhile, bring all these elements together, offering a unified view of data that supports informed decision-making at all levels.

With the serious shortage of doctors and other skilled health workers due to their migration from Africa to wealthier countries and the negative impact of this trend on healthcare access, the region faces an existential health crisis that requires the adoption of innovative strategies for improving the M&E of DHIs. By embracing these innovative strategies, we can significantly enhance the M&E of DHIs in sub-Saharan Africa. This will not only ensure the effective implementation of these interventions but also contribute to the broader goal of improving health access and outcomes across the region.

## References

[1] Kipruto H Muneene D Droti B Jepchumba V Okeibunor CJ Nabyonga-Orem J Use of digital health interventions in sub-saharan africa for health systems strengthening over the last 10 years: a scoping review protocol. Front Digit Health. 2022;4:874251. 10.3389/fdgth.2022.874251 35601887 PMC9120370

[2] Eddy-Spicer D Ehren M Bangpan M Monitoring and data use in developing countries: Findings from a systematic literature review. J Prof Cap Community. 2019;4:172–97. 10.1108/JPCC-11-2018-0028

[3] Enam A Torres-Bonilla J Eriksson H Evidence-based evaluation of eHealth interventions: systematic literature review. J Med Internet Res. 2018;20:e10971. 10.2196/10971 30470678 PMC6286426

[4] Prennushi G, Rubio G, Subbarao K. In: Monitoring and evaluation. A sourcebook for poverty reduction strategies. Washington D.C., USA: World Bank Group; 2002.

[5] Karamagi HC Muneene D Droti B Jepchumba V Okeibunor JC Nabyonga J eHealth or e-Chaos: The use of Digital Health Interventions for Health Systems Strengthening in sub-Saharan Africa over the last 10 years: A scoping review. J Glob Health. 2022;12:04090. 10.7189/jogh.12.04090 36462201 PMC9718445

[6] Etoori D Wringe A Kabudula CW Renju J Rice B Gomez-Olive FX Misreporting of patient outcomes in the South African national HIV treatment database: consequences for programme planning, monitoring, and evaluation. Front Public Health. 2020;8:100. 10.3389/fpubh.2020.00100 32318534 PMC7154050

[7] Azevedo MJ. The state of health system(s) in Africa: challenges and opportunities. In: Azevedo MJ, editor. Historical Perspectives on the State of Health and Health Systems in Africa, Volume II: The Modern Era. Cham, Switzerland: Springer; 2017. p. 1–73.

[8] Bervell B Al-Samarraie H A comparative review of mobile health and electronic health utilization in sub-Saharan African countries. Soc Sci Med. 2019;232:1–16. 10.1016/j.socscimed.2019.04.024 31035241

[9] Holst C Sukums F Radovanovic D Ngowi B Noll J Winkler AS Sub-Saharan Africa – the new breeding ground for global digital health. Lancet Digit Health. 2020;2:e160–2. 10.1016/S2589-7500(20)30027-3 33328076

[10] Otorkpa OJ Otorkpa C Adebola OA Emmanuel S Adamu A Olaniyan OE From Policy to Practice: A Review of Africa’s Public Health Policy. Cent Afr J Public Health. 2024;10:90–9. 10.11648/j.cajph.20241002.14

